# A novel functional module detection algorithm for protein-protein interaction networks

**DOI:** 10.1186/1748-7188-1-24

**Published:** 2006-12-05

**Authors:** Woochang Hwang, Young-Rae Cho, Aidong Zhang, Murali Ramanathan

**Affiliations:** 1 Department of Computer Science and Engineering, State University of New York at Buffalo, USA; 2 Department of Pharmaceutical Sciences, State University of New York at Buffalo, USA

## Abstract

**Background:**

The sparse connectivity of protein-protein interaction data sets makes identification of functional modules challenging. The purpose of this study is to critically evaluate a novel clustering technique for clustering and detecting functional modules in protein-protein interaction networks, termed STM.

**Results:**

STM selects representative proteins for each cluster and iteratively refines clusters based on a combination of the signal transduced and graph topology. STM is found to be effective at detecting clusters with a diverse range of interaction structures that are significant on measures of biological relevance. The STM approach is compared to six competing approaches including the maximum clique, quasi-clique, minimum cut, betweeness cut and Markov Clustering (MCL) algorithms. The clusters obtained by each technique are compared for enrichment of biological function. STM generates larger clusters and the clusters identified have p-values that are approximately 125-fold better than the other methods on biological function. An important strength of STM is that the percentage of proteins that are discarded to create clusters is much lower than the other approaches.

**Conclusion:**

STM outperforms competing approaches and is capable of effectively detecting both densely and sparsely connected, biologically relevant functional modules with fewer discards.

## Background

Since the first complete genome was sequenced in 1995, more than 300 prokaryotic genomes and more than 20 eukaryotic genomes have been sequenced [1]. Discovering the functional roles of gene products after the completion of sequencing the *Saccharomyces Cerevisiae *genome has been in the spotlight of post-genomic era. High-throughput techniques [2-5] for protein-protein interactions (PPI) detection have attracted researchers' attention since interacting proteins are likely to serve together as a group in cellular functions [6]. In recent years, high-throughput techniques in a genomic scale such as yeast-two-hybrid, mass spectrometry, and protein chip technologies have multiplied the volume of protein interaction datasets exponentially and also have provided us a genomic level view of molecular interactions. The cumulative PPI dataset of, for example, *S. Cerevisiae *in DIP (Database of Interacting Proteins) [7] now lists over 4900 proteins and 18,000 interactions from over 22000 experiments; however, nearly half of the proteins remain unannotated. Effective computational systems for storage, management, visualization and analysis are necessary to cope with these fast growing complex datasets.

PPI data provide us the good opportunity to systematically analyze the structure of a large living system and also allow us to use it to understand essential principles like essentiality, genetic interactions, functions, functional modules, protein complexes and cellular pathways. Cellular functions and biochemical events are coordinately carried out by groups of proteins interacting each other in functional modules, and the modular structure of complex networks is critical to function [6,8,9]. Identifying such functional modules in PPI networks is very important for understanding the structure and function of these fundamental cellular networks. Therefore, developing an effective computational approach to identify functional modules should be highly challenging but indispensable. Clustering analysis helps us understand the topological structure of the PPI networks and relationships among its components better. And, the principal function of each cluster can be inferred from the functions of its member. Functions for unannotated members of a cluster can be predicted by the functions of other annotated members [10].

PPI adjacency matrices can be represented as graphs whose nodes represent proteins and edges represent interactions. The clustering of a PPI dataset can be thereby reduced to graph theoretical problems. But, the binary nature of the current PPI data sets imposes challenges in clustering using conventional approaches. In the maximal clique approach, clustering is reduced to identifying fully connected subgraphs in the graph [11]. To overcome the relatively high stringency imposed by the maximal clique method, the Quasi Clique [7], Molecular Complex Detection (MCODE) [12], Spirin and Mirny [11] algorithms identify densely connected subgraphs rather than fully connected ones by either optimizing an objective density function or using a density threshold. The Restricted Neighborhood Search Clustering Algorithm (RNSC) [13] and Highly Connected Subgraphs (HCS) algorithms [14] harness minimum cost edge cuts for cluster identification. The Markov Cluster Algorithm (MCL) algorithm finds clusters using iterative rounds of expansion and inflation that promote the strongly connected regions and weaken the sparsely connected regions, respectively [15]. The line graph generation approach [9] transforms the network of proteins connected by interactions into a network of connected interactions and then uses the MCL algorithm to cluster the interaction network. Samantha and Liang [16] employed a statistical approach to clustering of proteins based on the premise that a pair of proteins sharing a significantly larger number of common neighbors will have high functional similarity.

However, currently used approaches encounter challenges because the relationship between protein function and PPI is characterized by weak connectivity and unexpected topological phenomena, such as low intraconnectivity and longish shapes of actual topological shapes of MIPS functional categories [17]. In our experimental analysis, subgraphs of each functional categories in MIPS database [17] are extracted from the Yeast PPI network, and the density of each subgraph is measured by Equation 7. The density of those subgraphs is averaged about 0.0023 which is fairly lower than we expected. Most functional categories have low connectivity within them in the PPI network and the majority of members in functional categories do not have direct physical interaction with other members of the functional category they belong to. Furthermore, it is not difficult to find singletons in the extracted subgraphs of functional categories which means that some proteins do not have any interaction with other proteins in the functional category they belong to. Let the diameter of a graph be the length of the longest path among all pair shortest paths in the graph. The average diameter of the subgraphs of all MIPS functional categories is approximately 4 which is close to the average shortest paths length, 5.47, of the whole PPI network. In other words, the subgraphs of actual MIPS functional categories in the PPI network generally are not closely congregated as we expected, they have longish shapes. Due to such low density within the modules, the existing approaches have produced a number of clusters with small size and singletons and mercilessly discarded many weakly connected nodes since they can only handle the strongly connected regions. Such incompleteness of clustering is a serious drawback of the existing algorithms. Discarding the sparsely connected nodes could be a hazardous loss of important information of the PPI network.

Biological networks, including PPI networks, illustrate the biochemical relationships of components in biochemical processes. Clustering of biological networks should be able to identify clusters of any arbitrary shapes and any density if the members of a cluster share important biochemical properties from the point of view of biochemical processes. To cope with this necessity and overcome those drawbacks of existing approaches, we propose a novel strategy to statistically analyze the degree of biological and topological influence of each protein to other proteins in a PPI network. We model PPI networks as a dynamic signal transduction system (STM) and demonstrate the signal transduction behavior of the perturbation by each protein on PPI networks statistically. This behavior should also reflect the topological properties of the network. The overall signal transduction behavior function between any two proteins will be formulated to evaluate the perturbation caused by a protein on other proteins biologically and topologically in the network. STM successfully identified the clusters with bigger size, arbitrary shape, low density, and biologically more enriched than other existing approaches did.

## 1 Method

### 1.1 The signal transduction model

The proteins and the protein-protein interactions in a PPI data set were, respectively, represented by nodes and edges of a graph. The graph representation was modeled using a pharmacodynamic signal transduction network model. Specifically, the signal transduction behavior of the network was modeled using the Erlang distribution, a special case of the Gamma distribution.

#### Graph definitions

An undirected graph *G *= (*V, E*) consists of a set *V *of nodes and a set *E *of edges, *E *⊆ *V *× *V*. An edge *e *= (*i, j*) connects two nodes *i *and *j*, *e *∈ *E*. The neighbors *N*(*i*) of node *i *are defined to be the set of directly connected nodes of node *i*. The degree *d*(*i*) of a node *i *is the number of the edges connected to node *i*. A path is defined as a sequence of nodes (*i*_1_,..., *i*_*k*_) such that from each of its nodes there is an edge to the successor node. The length of a path is the number of nodes in its node sequence. A shortest path between two nodes, *i *and *j*, is a minimal length path between them. The distance between two nodes, *i *and *j*, is the length of its shortest path.

#### Signal transduction model

We propose to model the dynamic relationships between proteins in a PPI network using a signal transduction network model. Specifically, the signal transduction behavior of the network is modeled using the Erlang distribution, a special case of the Gamma distribution. The Erlang distribution function is:

F(c)=1−e−xb∑k=0c−1(xb)kk!     (1)
 MathType@MTEF@5@5@+=feaafiart1ev1aaatCvAUfKttLearuWrP9MDH5MBPbIqV92AaeXatLxBI9gBaebbnrfifHhDYfgasaacH8akY=wiFfYdH8Gipec8Eeeu0xXdbba9frFj0=OqFfea0dXdd9vqai=hGuQ8kuc9pgc9s8qqaq=dirpe0xb9q8qiLsFr0=vr0=vr0dc8meaabaqaciaacaGaaeqabaqabeGadaaakeaacqWGgbGrcqGGOaakcqWGJbWycqGGPaqkcqGH9aqpcqaIXaqmcqGHsislcqWGLbqzdaahaaWcbeqaaiabgkHiTmaalaaabaGaemiEaGhabaGaemOyaigaaaaakmaaqahabaWaaSaaaeaadaqadaqaamaalaaabaGaemiEaGhabaGaemOyaigaaaGaayjkaiaawMcaamaaCaaaleqabaGaem4AaSgaaaGcbaGaem4AaSMaeiyiaecaaaWcbaGaem4AaSMaeyypa0JaeGimaadabaGaem4yamMaeyOeI0IaeGymaedaniabggHiLdGccaWLjaGaaCzcamaabmaabaGaeGymaedacaGLOaGaayzkaaaaaa@4DA8@

where *c *> 0 is the shape parameter, *b *> 0 is the scale parameter, *x *≥ 0 is the independent variable, usually time. The Erlang distribution has several characteristics, which are appropriate for describing the protein-protein interaction network, including its positive range and its important reproductive property [18]. The Erlang distribution with *x/b *= 1 is used and the value of *c *is set to the number of edges between source protein node and the target protein node. Setting the value of *x/b *to unity assesses the perturbation at the target protein when the perturbation reaches 1/*e *of its initial value at the nearest neighbor of the source protein node.

Erlang distribution models have been used in pharmacodynamics to model signal transduction and transfer delays in a variety of systems including the production of drug induced mRNA and protein dynamics [19] and calcium ion-mediated signaling in neutrophils [20]. The use of the Erlang distribution was motivated by several key physicochemical considerations. In formulating this framework, we noted that sequential cascades of protein-protein interactions are frequently observed in biological signal transduction processes.

**Figure 1:**
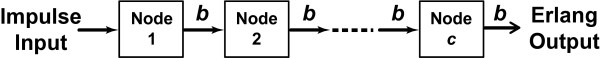
**The pharmacodynamic signal transduction model**. The pharmacodynamic signal transduction model whose impulse response is an Erlang distribution. The *b *is the time constant for signal transfer and *c *is the number of compartments.

In queuing theory, the distribution of time to complete a sequence of tasks in a system with Poisson input is described by the Erlang distribution. Because biological signal transduction can be modeled as a sequence of protein-protein interactions, we sought to apply these queuing results to PPI network modeling. The Erlang distribution also arises naturally in pharmacodynamics, where it has been used to effectively describe the dynamics of signal transduction in systems involving a series of protein compartments, e.g., in response to an unit impulse at time *t = *0, the signal transduction from the compartmental model in Figure 1 is equivalent to Erlang distribution. The Erlang distribution is a special case of the Gamma distribution and the latter has been shown to describe population abundances fluctuating around equilibrium [21]; this finding is relevant because perturbations to PPI networks will likewise cause alterations in the levels of bound and unbound protein complexes. Thus, we identified the Erlang distribution as a parsimonious model for describing the dynamics of PPI interactions.

The Erlang distribution needs to be further modified to reflect network topology. The perturbation induced by the source protein node should be proportional to its degree and to follow the shortest path to the target protein node. During transduction to the target protein node, the perturbation should dissipate at each intermediate visiting node to each incident edge. The signal transduced from node *v *to node *w *(*v *≠ *w*) is thus:

S(v→w)=d(v)∏i∈P(v,w)d(i)F(c)     (2)
MathType@MTEF@5@5@+=feaafiart1ev1aaatCvAUfKttLearuWrP9MDH5MBPbIqV92AaeXatLxBI9gBaebbnrfifHhDYfgasaacH8akY=wiFfYdH8Gipec8Eeeu0xXdbba9frFj0=OqFfea0dXdd9vqai=hGuQ8kuc9pgc9s8qqaq=dirpe0xb9q8qiLsFr0=vr0=vr0dc8meaabaqaciaacaGaaeqabaqabeGadaaakeaacqWGtbWucqGGOaakcqWG2bGDcqGHsgIRcqWG3bWDcqGGPaqkcqGH9aqpdaWcaaqaaiabdsgaKjabcIcaOiabdAha2jabcMcaPaqaamaarababaGaemizaqMaeiikaGIaemyAaKMaeiykaKcaleaacqWGPbqAcqGHiiIZcqWGqbaucqGGOaakcqWG2bGDcqGGSaalcqWG3bWDcqGGPaqkaeqaniabg+GivdaaaOGaemOrayKaeiikaGIaem4yamMaeiykaKIaaCzcaiaaxMaadaqadaqaaiabikdaYaGaayjkaiaawMcaaaaa@5189@

where *d*(*i*) is the degree of node *i, P*(*v, w*) is the set of the all nodes visited en route on the shortest path from node *v *to node *w*, excluding the source node *v *and the target destination node *w*, and *F *(*c*) is the signal transduction behavior function. When *v *= *w *and *distance *(*v, w*) = 0, we define *S *(*v *→ *w*) = *d *(*v*). The numerator of the first term in the right hand side of Equation 2 represents the degree of the source node *v*, and the denominator represents the dissipation on each visiting node on the shortest path from source node *v *to target node *w*. Our choice of the shortest path is motivated by the finding that the majority of flux prefers the path of least resistance in many physicochemical and biological systems. There can be more than one shortest path between a node pair in a network. STM chooses the least resistant path, which has the lowest resistance calculated by ∏_*i *∈ *P *(*v*, *w*) _*d *(*i*) in Equation 2, out of several tying shortest paths if there are more than one shortest path between a node pair. There also can be more than one least resistant path among several tying shortest paths. Choosing any one path out of several tying least resistant paths makes no difference in measuring the signal transduction quantity as long as it is a least resistant path since the signal quantity computed by Equation 2 depends only on the resistance not on any other topological properties of intermediate visiting nodes on a path. So, the first term in the right hand side of Equation 2 represents the topological effect of source node *v *on target node *w*. The second term in the right hand side of Equation 2 represents the biological effect of source node *v *on target node *w *in the signal transduction view point. Therefore, the nodes that score the highest value on target node *w *will be the most influential nodes on node *w *biologically and topologically.

**Figure 2:**
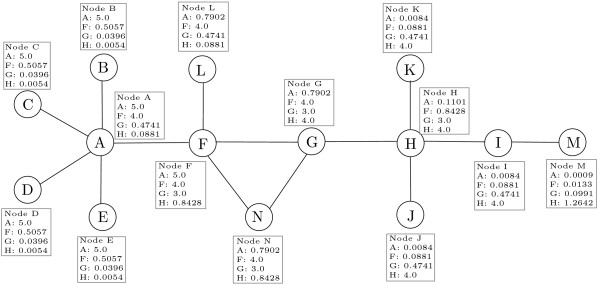
**A simple network example**. Each box contains the numerical values obtained from Equation 2 from nodes A, F, G, and H to other target nodes. Results for other nodes are not shown.

Figure 2 demonstrates the signal transduction behavior of a small example network according to Equation 2. For the ease of understanding, only the signals from node A, F, G, and H are presented, although signals should be propagated from each node in the network. Each box in Figure 2 contains the signal assessed by the Equation 2 from nodes A, F, G, and H to other target nodes, e.g., 5.0, 0.5057, 0.0396, 0.0054 are the signals assessed from nodes A, F, G, and H, respectively, on node E. These numerical values illustrate overall effects of combining the network topology with the signal transduction model from source nodes A, F, G, and H on node E. Consequently, node A, which has scored the highest value, will be the most influential node on node E biologically and topologically.

### 1.2 Clustering model

STM algorithm simulates the perturbation from each node to the other nodes in a network using Equation 2, which reflects the biological and topological properties of the node. Module representatives are the nodes that record the highest scores by Equation 2 on every node in a module, i.e., they are the most influential nodes in a module biologically and topologically. After the signal transduction simulation, each node selects the most influential nodes as the representatives of modules. From these representatives, preliminary modules can be formed by aggregating each node into each module that each of its representatives stands for. Finally, these preliminary modules are merged if there are substantial interconnections between them.

The pseudocode for the STM algorithm, which employs the signal transduction function of Equation 2 and a democratic representatives selection algorithm, is shown in Table 1. The algorithm involves four sequential processes:

**Table 1:** STM algorithm

**Algorithm 1: STM(G)**
1: V: set of nodes in Graph G
2: F(c): Transduction behavior function
3: S(*v, w*): arrived signal from node *v *to node *w*
4: C: the list of final clusters
5: PreClusters: the list of preliminary clusters
6: **for **each node *pair*(*v*, *w*) *v*, *w *∈ *V*, *v *≠ *w ***do**
7: distance(*v*, *w*) ← the shortest path length from node *v *to node *w*
8: set parameter *c *in function as F(c) as distance(*v*, *w*)
9: signal(*v, w*) ← S(v→w)=d(v)∏i∈P(v,w)d(i)F(c) MathType@MTEF@5@5@+=feaafiart1ev1aaatCvAUfKttLearuWrP9MDH5MBPbIqV92AaeXatLxBI9gBaebbnrfifHhDYfgasaacH8akY=wiFfYdH8Gipec8Eeeu0xXdbba9frFj0=OqFfea0dXdd9vqai=hGuQ8kuc9pgc9s8qqaq=dirpe0xb9q8qiLsFr0=vr0=vr0dc8meaabaqaciaacaGaaeqabaqabeGadaaakeaacqWGtbWucqGGOaakcqWG2bGDcqGHsgIRcqWG3bWDcqGGPaqkcqGH9aqpdaWcaaqaaiabdsgaKjabcIcaOiabdAha2jabcMcaPaqaamaarababaGaemizaqMaeiikaGIaemyAaKMaeiykaKcaleaacqWGPbqAcqGHiiIZcqWGqbaucqGGOaakcqWG2bGDcqGGSaalcqWG3bWDcqGGPaqkaeqaniabg+GivdaaaOGaemOrayKaeiikaGIaem4yamMaeiykaKcaaa@4DCA@
10: **end for**
11: **for **each node *v *∈ *V ***do**
12: *v*. representative ← select the best scored node *w *for node *v*
13: **if **cluster_*w *== null **then**
14: make cluster_*w*
15: cluster_*w*.add(*v*)
16: PreClusters.add(cluster_*w*)
17: **else**
18: cluster_*w*.add(*v*)
19: **end if**
20: **end for**
21: **C **← **Merge**(PreClusters)

**Process 1: **Compute signals transduced between all node pairs.

**Process 2: **Select cluster representatives for each node.

**Process 3: **Formation of preliminary clusters.

**Process 4: **Merge preliminary clusters.

Process 1 propagates signals from each source node and records the signal quantities on each target node for all node pairs according to Equation 2. The implementation of Process 1 is shown on lines 6–10 of the STM algorithm in Table 1.

In Process 2, each node elects the nodes from which it receives the highest signal value as the representatives of the clusters that the node will belong to. For example, in Figure 2, nodes A, B, C, D, E, and F will choose node A and nodes L, G, and N will choose node F, which are the best scored nodes on those nodes, as the representatives.

Each preliminary cluster is initialized by taking its representative as its initial member. Preliminary clusters are then augmented by accumulating each node toward the representatives chosen by each node. Lines from 11–20 in Table 1 contain the representative selection process and the preliminary cluster formation process. Notice that STM allows overlaps among clusters by opening the possibility of multiple representatives which have the tie score on a node, etc. For example, node G picks nodes F and H, which have the tie score on node G, as its representatives in Figure 2. Then, G will belong to the cluster formed by nodes F and the cluster formed by H. Therefore, overlaps occur between the cluster formed by node F, {F, G, L, N}, and the cluster formed by node H, {G, H, I, J, K, M}. STM identified three preliminary clusters, {A, B, C, D, E, F}, {F, G, L, N}, and {G, H, I, J, K, M}, based on the choice of representatives in Figure 2.

So far, STM considers only the biological influence and the least resistance paths between protein pairs in a network. Density, i.e., interconnectivity and intraconnectivity, of detected clusters should be another important aspect that we need to consider in modularization since the clusters that have high interconnections between them have high possibility being in the same functional module. In the final merge process described in Table 2, STM takes density among detected preliminary clusters into consideration by utilizing interconnectivity among detected preliminary clusters. Some preliminary clusters should be merged if they have substantial number of interconnections to improve clusters' quality. We propose to measure the degree of interconnectivity between clusters by the similarity of two clusters *i *and *j *defined below:

**Table 2:** Procedure: Merge(C)

1: C: the cluster list
2: MaxPair: the cluster pair(*c, k*) with max interconnections among all cluster pair
3: Max.value: interconnections between cluster pair *c *and *k*
4: MaxPair ← findMaxPair(C,null)
5: **while **Max.value ≥ **do**
6: newCluster ← merge MaxPair *c *and *k*
7: Replace cluster *c *with newCluster
8: Remove cluster *k*
9: MaxPair ← findMaxPair(C,newCluster)
10: **end while**
11: **return C**

Similarity(i,j)=interconnectivity(i,j)minsize(i,j)     (3)
 MathType@MTEF@5@5@+=feaafiart1ev1aaatCvAUfKttLearuWrP9MDH5MBPbIqV92AaeXatLxBI9gBaebbnrfifHhDYfgasaacH8akY=wiFfYdH8Gipec8Eeeu0xXdbba9frFj0=OqFfea0dXdd9vqai=hGuQ8kuc9pgc9s8qqaq=dirpe0xb9q8qiLsFr0=vr0=vr0dc8meaabaqaciaacaGaaeqabaqabeGadaaakeaacqWGtbWucqWGPbqAcqWGTbqBcqWGPbqAcqWGSbaBcqWGHbqycqWGYbGCcqWGPbqAcqWG0baDcqWG5bqEcqGGOaakcqWGPbqAcqGGSaalcqWGQbGAcqGGPaqkcqGH9aqpdaWcaaqaaGqaciab=LgaPjab=5gaUjab=rha0jabdwgaLjabdkhaYjabdogaJjabd+gaVjabd6gaUjabd6gaUjabdwgaLjabdogaJjabdsha0jabdMgaPjabdAha2jabdMgaPjabdsha0jabdMha5jabcIcaOiabdMgaPjabcYcaSiabdQgaQjabcMcaPaqaaiab=1gaTjab=LgaPjab=5gaUjabdohaZjabdMgaPjabdQha6jabdwgaLjabcIcaOiabdMgaPjabcYcaSiabdQgaQjabcMcaPaaacaWLjaGaaCzcamaabmaabaGaeG4mamdacaGLOaGaayzkaaaaaa@7054@

where *interconnectivity *(*i, j*) is the number of connections between clusters *i *and *j*, and *minsize*(*i, j*) is the size of the smaller cluster among clusters *i *and *j*. The *Similarity*(*i, j*) between two clusters *i *and *j *is the ratio of the number of the connections between them to the size of the smaller cluster. Highly interconnected clusters are iteratively merged based on the similarity of the clusters. The pair of clusters that have the highest similarity are merged in each iteration and the merge process iterates until the highest similarity of all cluster pairs is less than a given threshold. The selection of the threshold for merging clusters is a critical factor for the final cluster outcome. We can see that every member of the smaller cluster has at least one interaction with the members of the other cluster if *inter connectivity *(*i, j*) ≥ *minsize*(*i, j*) between cluster pair *i *and *j*. Therefore, we conclude that two clusters should be in the same functional module if every member of the smaller cluster has at least one interaction with the members of the other cluster. Theoretically and experimentally, we can see when *interconnectivity *(*i, j*) ≥ *minsize*(*i, j*), clusters *i *and *j *have substantial interconnections. Three clusters, {A, B, C, D, E, F}, {F, G, L, N}, {G, H, I, J, K, M}, are obtained after the Process 4 when 2.0 is used as the merge threshold. Two clusters, {A, B, C, D, E, F, G, L, N}, {G, H, I, J, K, M}, are obtained after the Merge process when 1.0 is used as the merge threshold.

### 1.3 Cluster assessment

The structures of the clusters identified by STM and other competing alternative approaches are assessed using several metrics.

The clustering coefficient, *C*(*v*), of a node *v *measures the connectivity among its direct neighbors:

C(v)=2|∪i,j∈N(v)(i,j)|d(v)(d(v)−1)     (4)
 MathType@MTEF@5@5@+=feaafiart1ev1aaatCvAUfKttLearuWrP9MDH5MBPbIqV92AaeXatLxBI9gBaebbnrfifHhDYfgasaacH8akY=wiFfYdH8Gipec8Eeeu0xXdbba9frFj0=OqFfea0dXdd9vqai=hGuQ8kuc9pgc9s8qqaq=dirpe0xb9q8qiLsFr0=vr0=vr0dc8meaabaqaciaacaGaaeqabaqabeGadaaakeaacqWGdbWqcqGGOaakcqWG2bGDcqGGPaqkcqGH9aqpdaWcaaqaaiabikdaYmaaemaabaWaambeaeaacqGGOaakcqWGPbqAcqGGSaalcqWGQbGAcqGGPaqkaSqaaiabdMgaPjabcYcaSiabdQgaQjabgIGiolabd6eaojabcIcaOiabdAha2jabcMcaPaqab0GaeSOkIufaaOGaay5bSlaawIa7aaqaaiabdsgaKjabcIcaOiabdAha2jabcMcaPiabcIcaOiabdsgaKjabcIcaOiabdAha2jabcMcaPiabgkHiTiabigdaXiabcMcaPaaacaWLjaGaaCzcamaabmaabaGaeGinaqdacaGLOaGaayzkaaaaaa@567E@

In Equation 4, *N *(*v*) is the set of the direct neighbors of node *v *and *d *(*v*) is the number of the direct neighbors of node *v*. Highly connected nodes have high values of clustering coefficient.

Degree centrality orders nodes by the number of their direct neighbors, and betweenness centrality measures the nodes' importance from the information flow point of view in a network. Degree and betweenness centrality commonly used to measure the importance of a node in a network. The Betweeness Centrality, *C*_*B *_(*v*), is a measure of the global importance of a node that assesses the proportion of shortest paths between all node pairs that pass through the node of interest. The Betweeness Centrality, *C*_*B *_(*v*) for a node of interest, *v*, is defined by:

CB(v)=∑s≠v≠t∈Vρst(v)ρst     (5)
 MathType@MTEF@5@5@+=feaafiart1ev1aaatCvAUfKttLearuWrP9MDH5MBPbIqV92AaeXatLxBI9gBaebbnrfifHhDYfgasaacH8akY=wiFfYdH8Gipec8Eeeu0xXdbba9frFj0=OqFfea0dXdd9vqai=hGuQ8kuc9pgc9s8qqaq=dirpe0xb9q8qiLsFr0=vr0=vr0dc8meaabaqaciaacaGaaeqabaqabeGadaaakeaacqWGdbWqdaWgaaWcbaGaemOqaieabeaakiabcIcaOiabdAha2jabcMcaPiabg2da9maaqafabaWaaSaaaeaaiiGacqWFbpGCdaWgaaWcbaGaem4CamNaemiDaqhabeaakiabcIcaOiabdAha2jabcMcaPaqaaiab=f8aYnaaBaaaleaacqWGZbWCcqWG0baDaeqaaaaaaeaacqWGZbWCcqGHGjsUcqWG2bGDcqGHGjsUcqWG0baDcqGHiiIZcqWGwbGvaeqaniabggHiLdGccaWLjaGaaCzcamaabmaabaGaeGynaudacaGLOaGaayzkaaaaaa@5089@

In the Equation 5, *ρ*_*st *_is the number of shortest paths from node *s *to *t *and *ρ*_*st *_(*v*) the number of shortest paths from *s *to *t *that pass through the node *v*.

The extent to which the clusters are associated with a specific biological function is evaluated using a p-value based on the hypergeometric distribution [7]. The p-value is the probability that a cluster would be enriched with proteins with a particular function by chance alone. The p-value is given by:

p=1−∑i=0k−1(Ci)(G−Cn−i)(Gn)     (6)
 MathType@MTEF@5@5@+=feaafiart1ev1aaatCvAUfKttLearuWrP9MDH5MBPbIqV92AaeXatLxBI9gBaebbnrfifHhDYfgasaacH8akY=wiFfYdH8Gipec8Eeeu0xXdbba9frFj0=OqFfea0dXdd9vqai=hGuQ8kuc9pgc9s8qqaq=dirpe0xb9q8qiLsFr0=vr0=vr0dc8meaabaqaciaacaGaaeqabaqabeGadaaakeaacqWGWbaCcqGH9aqpcqaIXaqmcqGHsisldaaeWbqaamaalaaabaWaaeWaaeaafaqabeGabaaabaGaem4qameabaGaemyAaKgaaaGaayjkaiaawMcaamaabmaabaqbaeqabiqaaaqaaiabdEeahjabgkHiTiabdoeadbqaaiabd6gaUjabgkHiTiabdMgaPbaaaiaawIcacaGLPaaaaeaadaqadaqaauaabeqaceaaaeaacqWGhbWraeaacqWGUbGBaaaacaGLOaGaayzkaaaaaaWcbaGaemyAaKMaeyypa0JaeGimaadabaGaem4AaSMaeyOeI0IaeGymaedaniabggHiLdGccaWLjaGaaCzcamaabmaabaGaeGOnaydacaGLOaGaayzkaaaaaa@4E0E@

In Equation 6, *C *is the size of the cluster containing *k *proteins with a given function; *G *is the size of the universal set of proteins of known proteins and contains *n *proteins with the function. Because the p-values are frequently small numbers with positive values between 0 and 1, the negative logarithms (to base 10, denoted -log p) are used. A -log p value of 2 or greater indicates statistical significance at *α *= 0.01.

The density of a subgraph *s *in a PPI network is measured by:

Ds=2en(n−1)     (7)
 MathType@MTEF@5@5@+=feaafiart1ev1aaatCvAUfKttLearuWrP9MDH5MBPbIqV92AaeXatLxBI9gBaebbnrfifHhDYfgasaacH8akY=wiFfYdH8Gipec8Eeeu0xXdbba9frFj0=OqFfea0dXdd9vqai=hGuQ8kuc9pgc9s8qqaq=dirpe0xb9q8qiLsFr0=vr0=vr0dc8meaabaqaciaacaGaaeqabaqabeGadaaakeaacqWGebardaWgaaWcbaGaem4Camhabeaakiabg2da9maalaaabaGaeGOmaiJaemyzaugabaGaemOBa4MaeiikaGIaemOBa4MaeyOeI0IaeGymaeJaeiykaKcaaiaaxMaacaWLjaWaaeWaaeaacqaI3aWnaiaawIcacaGLPaaaaaa@3CDF@

In Equation 7, *n *is the number of proteins and *e *is the number of interactions in a subgraph *s *of a PPI network.

## 2 Experimental results

### 2.1 Protein interaction data

The core data of *S. Cerevisiae *was obtained from the DIP database [22]. This dataset include 2526 proteins and 5949 filtered reliable physical interactions. Species such as *S. Cerevisae *provide important test beds for the study of the PPI networks since it is a well-studied organism for which most proteomics data is available for the organism, by virtue of the availability of a defined and relatively stable proteome, full genome clone libraries, established molecular biology experimental techniques and an assortment of well designed genomics databases [6,22].

### 2.2 Biological significance of the putative module representatives

Our signal transduction model of Equation 2 provides a vehicle to quantitatively measure the degree of biological and topological influence of each protein on other proteins in the PPI network. The most influential proteins, that is, the highest scored nodes, are highly important proteins. To evaluate the biological significance of the most influential proteins, we annotated the lethality of each protein in the yeast PPI network according to the MIPS lethality data. Lethality is a crucial factor to characterize the biological essentiality of a protein. It is determined by examining whether a module is functionally disrupted when the protein is knocked out. We obtained the protein lethality information from MIPS database [17], which reports whether a protein is lethal or viable. We found that 233 proteins out of the top scored 555 proteins are lethal.

**Figure 3:**
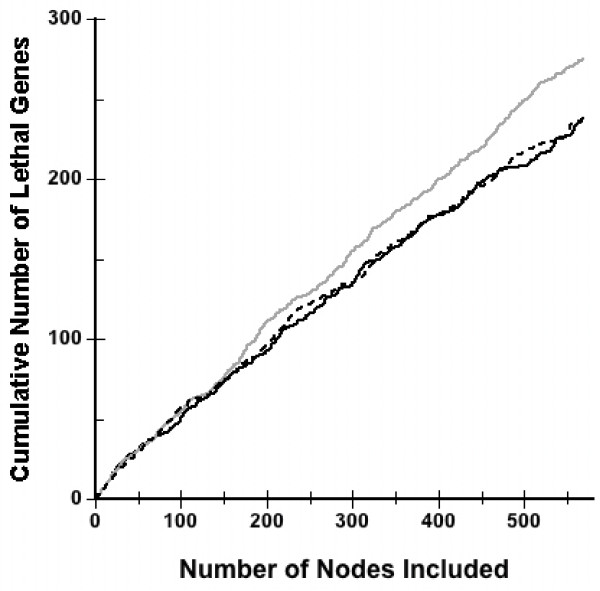
**Accumulation of lethal proteins for various percentiles**. Accumulation of lethal proteins for various percentiles of degree (gray line), betweeness centrality (dashed line) or the STM signal transduction metric (solid line). The results are shown for the top 555 proteins obtained from the yeast PPI network and are ordered; the highest values of these metrics are closest to the origin.

Figure 3 plots the cumulative number of lethal genes vs. the number of protein nodes included for increasing percentiles of the degree, betweeness or the STM signal transduction metric. The data are shown for 555 genes, obtained from the yeast PPI network, with the highest values of each of these metrics. In each case, the results are sorted and highest values are placed closest to the origin. Figure 3 shows that the performance of the STM metric in predicting lethality is comparable to that of degree and betweeness approaches for up to 150 nodes.

### 2.3 Clustering performance analysis

Experimentally, we performed STM algorithm on the yeast PPI data set using various merge threshold values to find the best threshold value for each data set. Experiments using 0.5,1.0,1.5, 2.0, 2.5, and 3.0 as the merge threshold were performed on each data set. The results show that when the merge threshold is less than 1.0, clusters that do not have substantial similarity are merged; and when the merge threshold is greater that 1.5, merging seldom occurred. There is no much performance difference when the values between 1.0 and 1.5 are used. The experiment when 1.0 is used as the merge threshold showed the best performance.

#### 2.3.1 Cluster analysis

*555 *preliminary clusters are obtained from the yeast PPI network and merged using 1.0 as the merge threshold. In Table 3, all 60 clusters that have more than 4 proteins are listed, and it also shows their topological characteristics and their assigned molecular functions from MIPS functional categories. To facilitate critical assessments, the percentage of proteins that are in concordance with the major assigned function (hits), the discordant proteins (misses) and un-known are also indicated. Among these 60 clusters, the largest one contains 210 proteins and the smallest one contains 5 in them. On average, we have 40.1 proteins in a cluster, and the average density of the subgraphs of the clusters extracted from the PPI network is 0.2145. The -log p values of the major function identified in each cluster is also shown and these values provide a measure of the relative enrichment of a cluster for a given functional category: higher values of -log p indicate greater enrichment. The results demonstrate that the STM method can detect large but sparsely connected clusters as well as small densely connected clusters. The high values of -log p (values greater than 2.0 indicate statistical significance at *α *< 0.01) indicate that clusters are significantly enriched for biological function and can be considered to be functional modules. As a result, our method can clearly identify larger modules that have low density but still biologically enriched as we can see from the size, the density, and the P-value of the clusters in Table 3.

**Table 3:** STM clustering result on the yeast PPI dataset

			Distribution				
					
Cluster	Size	Density	H	D	U	-Log*p*	Function
1	214	0.019	24.7	69.6	5.6	43.9	Nuclear transport
2	188	0.015	69.1	25.0	5.8	36.4	Cell cycle and DNA processing
3	181	0.022	22.0	72.3	5.5	17.2	Cytoplasmic and nuclear protein degradation
4	170	0.028	46.4	42.9	10.5	31.6	Transported compounds (substrates)
5	131	0.028	37.4	55.7	6.8	28.6	Vesicular transport (Golgi network, etc.)
6	125	0.030	60.8	33.6	5.6	32.2	tRNA synthesis
7	113	0.027	19.4	71.6	8.8	11.8	Actin cytoskeleton
8	79	0.045	17.7	73.4	8.8	12.3	Homeostasis of protons
9	78	0.033	26.9	62.8	10.2	12.5	Ribosome biogenesis
10	76	0.041	38.1	59.2	2.6	20.2	rRNA processing
11	72	0.030	5.6	84.7	9.7	6.2	Calcium binding
12	68	0.064	66.1	25.0	8.8	44.5	mRNA processing
13	61	0.041	40.9	52.4	6.5	11.5	Cytoskeleton
14	58	0.064	72.4	27.6	0.0	37.4	General transcription activities
15	53	0.048	15.0	71.6	13.2	7.9	MAPKKK cascade
16	50	0.064	66.0	32.0	2.0	33.5	rRNA processing
17	45	0.055	24.4	73.3	2.2	11.1	Metabolism of energy reserves
18	44	0.058	59.0	36.3	4.5	5.1	Metabolism
19	39	0.072	10.2	89.7	0.0	7.3	Cell-cell adhesion
20	36	0.125	58.3	36.1	5.5	16.9	Vesicular transport
21	29	0.091	55.1	44.8	0.0	8.3	Phosphate metabolism
22	28	0.074	14.2	78.5	7.1	4.5	Lysosomal and vacuolar protein degradation
23	27	0.119	29.6	66.6	3.7	7.3	Cytokinesis (cell division)/septum formation
24	26	0.153	53.8	46.1	0.0	28.6	Peroxisomal transport
25	25	0.090	28.0	68.0	4.0	4.6	Regulation of C-compound and carbohydrate utilization
26	25	0.116	68.0	28	4.0	12.9	Cell fate
27	22	0.151	59.0	36.3	4.5	11.4	DNA conformation modification
28	21	0.147	76.1	19.0	4.7	23.9	Mitochondrial transport
29	20	0.200	75.0	20.0	5.0	24.0	rRNA synthesis
30	19	0.228	78.9	15.7	5.2	17.9	Splicing
31	17	0.220	70.5	29.4	0.0	19.7	Microtubule cytoskeleton
32	17	0.183	23.5	76.4	0.0	8.2	Regulation of nitrogen utilization
33	15	0.304	86.6	13.3	0.0	31.3	Energy generation
34	14	0.142	50.0	42.8	7.1	9.0	Small GTPase mediated signal transduction
35	13	0.564	76.9	23.0	0.0	15.9	Mitosis
36	13	0.358	84.6	15.4	0.0	12.4	DNA conformation modification
37	13	0.410	69.2	23.0	7.6	17.6	3'-end processing
38	13	0.179	61.5	30.7	7.6	6.7	DNA recombination and DNA repair
39	12	0.196	16.6	75.0	8.3	3.9	Unspecified signal transduction
40	12	0.363	58.3	41.6	0.0	14.7	Posttranslational modification of amino acids
41	12	0.166	16.6	75.0	8.3	2.4	Autoproteolytic processing
42	11	0.218	54.5	45.4	0.0	2.9	Transcriptional control
43	11	0.200	72.7	27.2	0.0	8.2	Enzymatic activity regulation/enzyme regulator
44	10	0.466	80.0	20.0	0.0	14.8	Translation initiation
45	9	0.361	77.7	22.2	0.0	12.8	Translation initiation
46	8	0.321	50.0	37.5	12.5	5.6	Metabolism of energy reserves
47	8	0.321	75.0	25.0	0.0	9.0	Modification by ubiquitination, deubiquitination
48	8	0.321	37.5	62.5	0.0	3.7	Mitosis
49	7	0.333	42.8	57.1	0.0	3.5	DNA damage response
50	7	0.333	57.1	28.5	14.2	4.1	Vacuolar transport
51	7	0.285	28.5	71.4	0.0	4.4	Biosynthesis of serine
52	6	0.333	50.0	33.3	16.6	2.38	Modification by phosphorylation, dephosphorylation, etc.
53	5	0.400	100	0.0	0.0	7.0	Meiosis
54	5	0.600	100	0.0	0.0	7.0	Vacuolar transport
55	5	0.400	100	0.0	0.0	8.5	ER to Golgi transport
56	5	0.400	20.0	40.0	40.0	1.8	cAMP mediated signal transduction
57	5	0.500	40.0	40.0	20.0	3.1	Oxidative stress response
58	5	0.500	80.0	20.0	0.0	4.4	Intracellular signalling
59	5	0.600	40.0	60.0	0.0	4.2	Tetracyclic and pentacyclic triterpenes
60	5	0.400	60.0	40.0	0.0	4.1	Mitochondrial transport

The first column is a cluster identifier; the Size column indicates the number of proteins in each cluster; the Density indicates the density of the cluster; the H column indicates the percentage of proteins concordant with the major function indicated in the last column; the D column indicates the percentage of proteins discordant with the major function and U column indicates percentage of proteins not assigned to any function.

**Figure 4:**
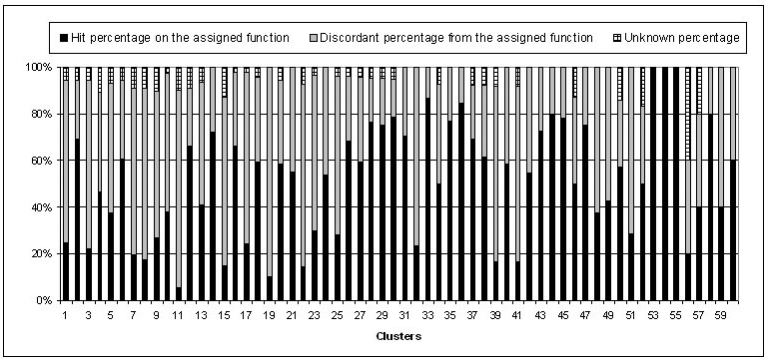
**Distribution of the three classes of 60 clusters**. Distribution of the three classes of 60 clusters: the hit percentage with the assigned function, discordant percentage from the assigned function, and unknown percentage.

Figure 4 exhibits the distribution of the hit, miss, and unknown percentage of member proteins with the assigned function for each cluster in Table 3 for better understanding visually. We found that most of the proteins in a cluster have the same functions that are assigned as a main function for the cluster as shown in Figure 4.

#### 2.3.2 Comparative analysis

The results in Table 4 and 5 for the yeast PPI dataset show that STM generates larger clusters; the clusters identified had p-values that are 2.2 orders of magnitude or approximately 125-fold lower than Quasi clique, the best performing alternative clustering method, on biological function. The p-values for the cellular localization are also shown in the last column of Table 4 and 5. It is clear that the clusters identified by STM despite being larger have low p-values. Although p-values generally decrease with increasing cluster size, these decreases in p-values can occur only when the null hypothesis is false. The p-values reflect the confidence that the differences, if present, are not due to chance alone. The confidence in any given result increases when these are obtained in a larger sample and in this context. So, the dependence of p-values on sample size is intuitive. The p-values express the strength of evidence against the null hypothesis to account for both the sample size, the amount of noise in measurements. Therefore, the STM clusters have low p-values because they are enriched for function and not simply because they are larger.

**Table 4:** Comparison of STM to competing clustering methods for clusters with 5 or more members

Method	Number	Size	Discard(%)	Function	Location
**STM**	**60**	**40.1**	**7.8**	**13.7**	**7.42**
Maximal clique	120	5.65	98.4	10.6	7.93
Quasi clique	103	11.2	80.8	11.5	6.58
Samantha	64	7.9	79.9	9.16	4.89
Minimum cut	114	13.5	35.0	8.36	4.75
Bwtweenness cut	180	10.26	21.0	8.19	4.18
MCL	163	9.79	36.7	8.18	3.97

Comparison of STM to competing clustering methods for the yeast protein-protein interaction data set for clusters with 5 or more members. The Number column indicates the number of clusters identified by each method, the Size column indicates the average number of proteins in each cluster; the Discard% indicates the percentage of proteins not assigned to any cluster. The -log p values for biological function and cellular location are shown.

**Table 5:** Comparison of STM to competing clustering methods for clusters with 9 or more members

Method	Number	Size	Discard(%)	Function	Location
**STM**	**45**	**52.4**	**11.5**	**16.8**	**9.01**
Maximal clique	N/A	N/A	N/A	N/A	N/A
Quasi clique	46	16.7	86.7	15.3	9.34
Samantha	17	12.3	93.3	15.9	7.65
Minimum cut	44	24.3	55.0	14.8	8.78
Bwtweenness cut	78	14.4	50.5	11.3	6.05
MCL	55	16.7	69.4	11.5	5.42

Comparison of STM to competing clustering methods for the yeast protein-protein interaction data set for clusters with 9 or more members. The Maximal clique does not identify clusters with 9 or more members. The footnote is the same to Table 4.

Tables 4 and 5 demonstrate that STM outperforms the other existing approaches. We made a comparison with 6 other existing approaches, Maximal cliques [11], Quasi cliques [7], Samantha [23], Minimum cut [18], Betweenness cut [24], and MCL [15]. The comparison on the cluster size more than 4 is in Table 4 and on the cluster size more than 9 in Table 5. Both tables show that our signal transduction model based method generates considerably larger clusters, and the identified clusters by our method have at least 2 orders of magnitude higher P-value than the others on both function and localization categories.

Quasi clique and Maximal clique discarded 80.8% and 98.4% nodes during clustering process, even though they identified the clusters with relatively high p-values in Table 4. Quasi clique and Samantha discarded 86.7% and 93.3% nodes, even though they identified the clusters with relatively high p-values in the clusters with size more than 9 in Table 5. Another important strength of STM is that the percentage of proteins that are discarded to create clusters is 7.8%, which is much lower than the other approaches, which have an average discard percentage of 59%. The yeast PPI dataset is relatively modular and the bottom-up approaches (e.g., maximal clique and quasi clique methods) generally outperformed the top-down approaches (exemplified by the minimum cut and betweeness cut methods) on functional enrichment as assessed by -log p. However because bottom-up approaches are based on connectivity of dense regions, the percentages of discarded nodes for the bottom-up methods are also higher than STM and the top-down approaches. But, we already have shown that the functional modules have fairly low density and arbitrary shapes with long diameter. So, discarding those sparsely connected proteins could be a fatal decision which might resulted in the important biological information losses. Consequently, STM is versatile and its performance on biological function and localization enrichment, cluster size, and discard rate is superior to the best of the other six methods on both data sets.

### 2.4 Computational complexity analysis

STM is fundamentally established on all pairs shortest path searching algorithm to measure the distance between all node pairs. This problem can be solved in *O *(*V*^2^*logV *+ *V E*) time if it is implemented using Johnson's algorithm [25], where *V *is the number of nodes and *E *is the number of edges in a graph. After measuring the distance between all node pairs, formation of preliminary clusters takes *O *(*V*) time. The amount of time required to find the best cluster pair that has the most interconnections is *O *(*k*^2 ^*logk*) by using heap-based priority queue, where *k *is the number of preliminary clusters [26]. The Merging process needs to find the cluster pair which has the most interconnections, and it takes *O *(*k*^2 ^*logk*) time only for the initial iteration. From the second iteration, finding the best cluster pair takes *O *(*klogk*) time since the cluster pair comparisons are needed only between the newly merged cluster and the other clusters. And the maximum *k*, the number of preliminary clusters, is at most *O *(*V*) in the case of the fully connected graph, therefore the Merging process takes *O *(*V*^2 ^*log V*) time. But *k *is much smaller than *V *in sparse networks like the Yeast PPI network. So the total time complexity of our algorithm is bounded by the time consumed in computing the distance between all node pairs, which is *O *(*V*^2 ^*log V *+ *V E*).

## 3 Discussion

We have studied that the topological shapes of the subgraphs of MIPS functional categories extracted from the PPI network are arbitrary and the density of them is fairly low. These two unexpected properties of functional categories prohibited other existing approaches from detecting functional modules from PPI networks effectively. A relative excess of emphasis on density and interconnectivity in the existing methods can be preferential for detecting clusters with relatively balanced round shapes and limit performance. The incompleteness of clustering is another distinct drawback of existing algorithms, which produce many clusters with small size and singletons. The preference for strongly connected nodes results in many weakly connected nodes being discarded. Moreover, considering only the topological properties and ignoring the biological characteristics of the network also can damage the effectiveness of clustering.

In this paper we have proposed a novel clustering method based on the signal transduction model for the Yeast PPI network. In head-to-head comparisons, the STM outperformed competing approaches and is capable of effectively detecting both dense and sparsely connected, biologically relevant functional modules with fewer discards. To our knowledge, this is the first description of the use of signal transduction based approach for this application.

Overwhelming performance of our approach has been demonstrated in several criteria including visual inspection. STM generated bigger size clusters with arbitrary shape, and those identified clusters are more biologically enriched, i.e., higher P-value, even though they have low density. There are more than 5% of unannotated proteins in the identified clusters. The function of those unannotated proteins can be predicted according to their assigned main functions by our method. Completeness of our clustering method is another distinct strength compared to the other methods. Our method discarded only about 7.8% of proteins which is tremendously lower than the other approaches did, 59% in average. In conclusion, STM has strong pharmacodynamics-based underpinnings and is an effective, versatile approach for analyzing protein-protein interactions. The STM approach contains a framework for rationally incorporating reaction rates, protein concentrations and interaction stoichiometry should these become available. It could therefore have potential applications in the drug discovery and development.
